# Biofilm-derived curli and Z-DNA shape anti-DNA antibody responses during Salmonella infections

**DOI:** 10.1371/journal.ppat.1014047

**Published:** 2026-07-14

**Authors:** Molly Elkins, Kaitlyn Grando, Courtney Covolo, Diane Spencer, Jacob F. Maziarz, Erin M. Vasicek, Carly DeAntoneo, Francisco J. Albicoro, Shingo Bessho, Zachary W. Reichenbach, Sophia Olubajo, Bettina Buttaro, Siddharth Balachandran, John S. Gunn, David Pisetsky, Çagla Tükel

**Affiliations:** 1 Center for Microbiology and Immunology, Temple University Lewis Katz School of Medicine, Philadelphia, Pennsylvania, United States of America; 2 Durham VA Medical Center and Duke University Medical Center, Durham, North Carolina, United States of America; 3 Center for Microbe and Immunity Research, Abigail Wexner Research Institute at Nationwide Children’s Hospital, Columbus, Ohio, United States of America; 4 Center for Immunology, Fox Chase Cancer Center, Philadelphia, Pennsylvania, United States of America; 5 Department of Medicine, Division of Gastroenterology, Temple University Hospital, Philadelphia, Pennsylvania, United States of America; 6 Center for Substance Abuse Research, Temple University Lewis Katz School of Medicine, Philadelphia, Pennsylvania, United States of America; 7 Sol Sherry Thrombosis Research Center, Temple University Lewis Katz School of Medicine, Philadelphia, Pennsylvania, United States of America; 8 Department of Pediatrics, College of Medicine, The Ohio State University, Columbus, Ohio, United States of America; UMass Chan Medical School, UNITED STATES OF AMERICA

## Abstract

Antibodies to Z-DNA, a non-canonical DNA conformation with a left-handed zigzag backbone, are abundant in the serum of patients with systemic lupus erythematosus (SLE), with levels increasing with disease activity and flares. As SLE is associated with bacterial infections, and as extracellular DNA (eDNA) within biofilms of several bacterial species has been shown to adopt the Z-DNA conformation, bacterial Z-DNA may represent a source of immunogenic Z-DNA in SLE and other related autoimmune conditions. In these studies, we investigated whether eDNA in Salmonella biofilms also contained Z-DNA and whether such Z-DNA could elicit an antibody response. Using antibody-based staining approaches, we observed abundant eDNA in *Salmonella enterica* serovar Typhimurium (STm) biofilms in both the Z- and canonical B-DNA configurations, consistent with the highly Z-prone nature of the GC-rich *Salmonella* genome. To assess the functional contribution of these DNA conformations to biofilm integrity, biofilms were treated with DNase I, which lacks enzymatic activity against Z-DNA, or with benzonase, a nonspecific nuclease that degrades both B- and Z-DNA. DNase I treatment applied after biofilm maturation was less effective at thinning biofilms than treatment during early biofilm formation, a pattern also observed with benzonase treatment. Purified curli:DNA complexes contained Z-DNA and, when administered intraperitoneally to mice, elicited robust anti-Z-DNA antibody responses. Similarly, infection with invasive STm induced the production of anti-Z-DNA antibodies *in vivo*. Moreover, STm infection in mice fed a diet that promotes biofilm development was associated with increased Z-DNA levels in the cecal lumen and elevated anti-DNA antibody responses. Collectively, these findings suggest that Z-DNA, likely formed by extruded *Salmonella* genomic DNA, and embedded within curli:DNA complexes of STm biofilms, triggers a host immune response and drives anti–Z-DNA antibody production. This work provides mechanistic insight into how bacterial infections and diet-dependent modulation of biofilm formation may contribute to anti-Z-DNA antibody responses in autoimmune diseases like SLE.

## Author summary

DNA is not always in the common right-handed double helix form; it can also flip into a rarer left-handed form known as Z-DNA, which has been linked to autoimmune responses. Studying biofilms formed by *Salmonella enterica* serovar Typhimurium, we found that extracellular DNA within these communities adopts both B- and Z-DNA forms. We show that this DNA, especially when bound to curli amyloid fiber, can stimulate strong anti–Z-DNA antibody responses in mice. Infection with Salmonella, particularly under conditions that promote biofilm formation, increased Z-DNA levels and antibody production in vivo. These findings suggest that bacterial biofilms may contribute to autoimmune responses by providing a previously unrecognized source of immunogenic Z-DNA.

## Introduction

Antibodies directed against DNA are observed in multiple autoimmune diseases; however, in systemic lupus erythematosus (SLE) [1], they are widely used as markers for disease classification and as biomarkers of disease activity [2,3]. In studies on the pathogenesis of SLE, the origin of anti-double-stranded DNA (dsDNA) antibody production has been particularly enigmatic, since immunization of animals with B-DNA, the classic right-handed, Watson-Crick base-paired, double-helical B conformation, fails to induce autoantibody production, even in the presence of carriers and adjuvants [3,4]. In contrast to B-DNA, certain DNA structures, such as Z-DNA, can also induce an immune response when administered to animals [5,6]. Z-DNA is a left-handed helix with a phosphodiester backbone in a zig-zag orientation. The Z-DNA conformation is energetically unfavorable, but alternating purine-pyrimidine sequences can adopt this conformation under certain environmental conditions. The transition from B-DNA to Z-DNA can be facilitated by base methylation or chemical modification (e.g., bromination of bases) [5,7–11]. Z-DNA can potently induce antibodies in animals [11–15], suggesting that, unlike B-DNA, Z-DNA is immunogenic and fails to induce immune tolerance [16]. Although antibodies to Z-DNA are found in SLE patients, the source of immunogenic Z-DNA has remained a mystery for decades, as it had been generally accepted that this type of DNA does not readily occur in nature, at least in mammalian organisms.

DNA is a major component of biofilms, which are multicellular communities of bacteria enclosed in an extracellular matrix (ECM). The ECM of biofilms is composed of nucleic acids, proteins, and carbohydrates that physically protect the bacteria from the environment including the immune system. The secreted protein curli accounts for about 85% of the ECM mass in *Salmonella enterica* serovar Typhimurium (STm) biofilms [17]. STm forms biofilms in the intestinal lumen and on gallstones [18–20]. It is believed that STm strategically employs two lifestyles, planktonic and biofilm, to establish a successful infection [18,21,22]. In mouse models, *Salmonella* infection induces a robust type I interferon (IFN) response [23] and leads to the production of anti-DNA autoantibodies [18,23–25]. Curli becomes hyperinflammatory when complexed with eDNA in bacterial biofilms, eliciting anti-DNA antibodies of the pathogenic IgG2b subtype and generation of type I IFNs and other pro-inflammatory cytokines [18,23,26–28].

Recent studies have demonstrated that bile acids and cholesterol play a central role in promoting *Salmonella* biofilm formation and curli production [19,29,30]. Consistent with these results, a study by Cruz-cruz *et al.* showed that mice fed a high-cholesterol lithogenic diet (LD), which increases intestinal bile acid concentration, exhibited increased STm biofilm formation and curli production in the gut [20]. Notably, Buzzo *et al.* recently showed that eDNA within the ECM of biofilms from *Haemophilus influenzae*, *Pseudomonas aeruginosa*, and uropathogenic *Escherichia coli* adopts a left-handed Z-DNA conformation as a result of interactions with DNA-binding proteins in the ECM [31]. However, whether STm biofilms similarly harbor Z-DNA is unknown; it is also unclear whether biofilm-associated Z-DNA could serve as a source of anti-Z-DNA immune responses during infection.

In this study, we demonstrate that STm biofilms contain Z-DNA, and characterize Z-DNA/curli interactions within the biofilm matrix. We then directly tested whether biofilms or biofilm components drive anti-Z-DNA production by determining whether *in vivo* administration of purified curli complexes containing Z-DNA or oral infection with STm induces anti–Z-DNA antibody production in mice. In parallel, we investigated whether enhancing STm biofilm formation through a lithogenic diet can amplify anti-Z-DNA antibody responses during infection. Together, these findings identify STm biofilms as a key source of Z-DNA, with curli serving as a structural scaffold that facilitates its presentation. Diet-induced enhancement of biofilm formation amplifies these responses, and complementary experiments reveal a mechanistic link between Z-DNA-producing biofilms, bacterial infection, and the development or exacerbation of anti-Z-DNA immune responses.

## Results

### Z-DNA is present in STm biofilms

**Fig 1:**
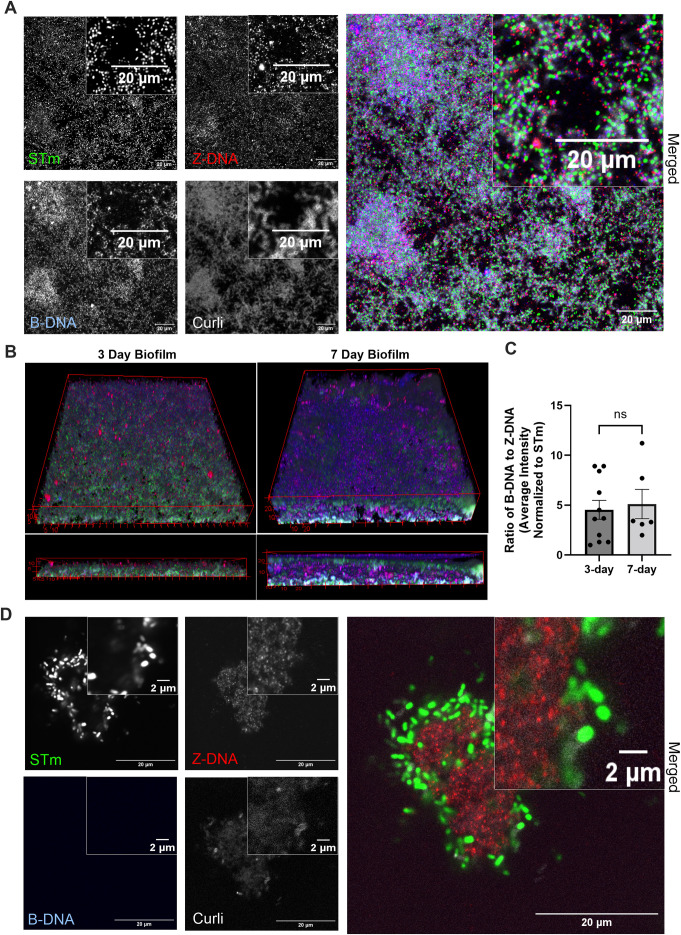
STm biofilms contain Z-DNA. STm was grown on glass cover slips at 28˚C for **(A-D)** 72 hours or **(B-C)** 7 days and stained with Syto9 dye showing bacterial cells (green), antibodies against Z-DNA (red) and B-DNA (blue), and FSB amyloid dye for curli (gray). Images were acquired at 100X using confocal microscopy and analyzed in ImageJ. Scale bars represent 20µm. **(C)** The mean fluorescence intensity (MFI) of B-DNA and Z-DNA was measured in ImageJ, the ratio of B- to Z-DNA was calculated, and normalized to the MFI of STm. The experiment was repeated at least three times. Representative images are shown. Each dot represents the average of three visual fields per one biofilm **(C)**.

While Z-DNA can provide structural integrity to certain human pathogenic bacterial biofilms [31], there has been no direct evidence of its role in STm biofilms prior to these experiments. To investigate the presence of eDNA in the Z-conformation in the STm ECM, we grew STm biofilms *in vitro* for 72 hours on glass slides. We performed immunofluorescence staining and imaged biofilms using confocal microscopy. Both B-DNA and Z-DNA were readily detected within the 3-day biofilm ECM, co-localizing with curli in discrete regions enriched for each DNA conformation (Fig 1A). We next examined 7-day-old mature biofilms, as prior studies have shown that Z-DNA content increases as the biofilm matures [31]. We again observed regions enriched for both B-DNA and Z-DNA (Fig 1B). Consistent with previous studies, the biofilms appeared visually thicker at 7 days [31]; however, the relative proportions of B-DNA and Z-DNA did not differ between the 3-day and 7-day time points (Fig 1C). We also noted regions of the biofilm in which Z-DNA and B-DNA were not both present; instead, Z-DNA was observed in the absence of B-DNA (Fig 1D) and in open spaces between cell clusters (Fig 1A). Together, these findings demonstrate that Z-DNA, a non-canonical eDNA, is present throughout STm biofilms and may associate with other ECM components during biofilm development.

### Z-DNA supports structural integrity and modulates biofilm robustness

**Fig 2:**
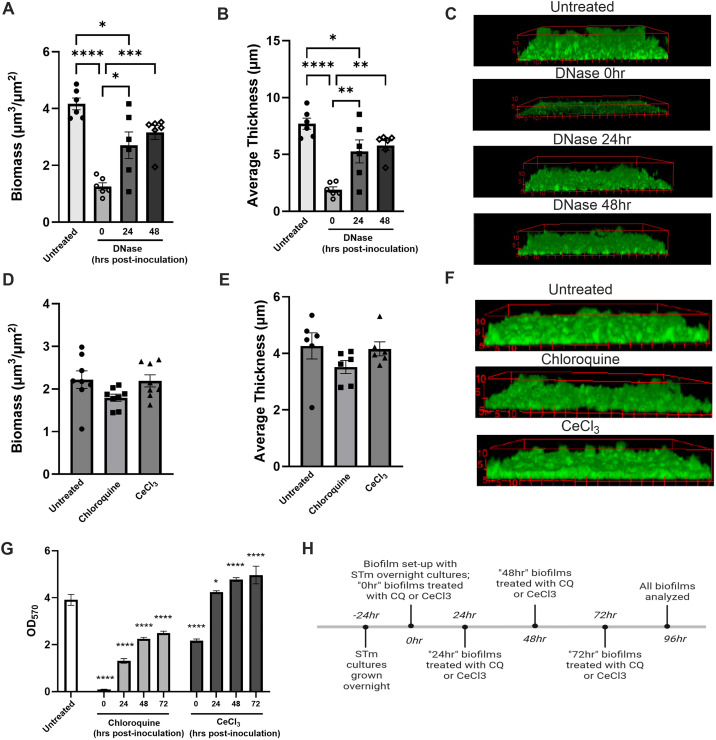
Z-DNA contributes to STm biofilm ECM architecture. **(A-C)** Biofilms were grown on glass chamber slides and treated with 10U/mL DNase at setup (0 hours), 24 hours, or 48 hours post-inoculation, stained with Syto60, imaged by confocal microscopy at 63X, and analyzed for (A) biomass measured as 3D volume μm^3^ over area μm^2^ (B) average thickness in μm for the entire area via Comstat2 within ImageJ. Multiple measurements were taken from each biofilm. Confocal images are shown in **(C)**. **(D-F)** Separate biofilms were treated with chloroquine or CeCl_3_ at 24 hours post-inoculation, then stained with Syto60, imaged via confocal microscopy at 96 hours, and measured for (D) biofilm biomass and (E) average thickness; (F) representative confocal images are shown. **(G)** Separate biofilms were treated with chloroquine or CeCl_3_ at setup (0 hours), 24 hours, 48 hours, or 72 hours post-inoculation and biofilm mass was analyzed via crystal violet assay. **(H)** Schematic diagram of chloroquine or CeCl_3_ treatments for crystal violet assay. *p < 0.05, **p < 0.01, ***p < 0.001, and ****p < 0.0001. For Fig 2G, comparisons were made to the untreated condition. Experiments were repeated at least three times. Representative images from one experiment is shown. Each dot represents the average of three visual fields per one biofilm (A,B,D,E).

To further evaluate the contribution of Z-DNA to biofilm structure, we performed DNase treatment assays. Because Z-DNA is inherently resistant to DNase I digestion while B-DNA is readily degraded [32], DNase treatment provides a useful approach to distinguish the proportions of these DNA conformations within the extracellular matrix, and to assess their contributions to the development of the biofilm. To assess the role of different DNA conformations, we added DNase I to STm biofilms at various time points and quantified total biomass using confocal microscopy and Comstat Biomass, which measures 3D physical volume (µm^3^) normalized to area (µm^2^) and biofilm thickness (µm). When DNase I was added at the very start of biofilm development (0 hour), we observed a marked reduction in both biomass and thickness, suggesting that DNase-sensitive eDNA (likely in the B-form) is important for initial biofilm establishment. By contrast, DNase treatment at later stages (24 and 48 hours post-inoculation) produced progressively smaller reductions in these measures (Fig 2A-2C). This pattern suggests that as biofilms mature, an increasing proportion of the eDNA adopts the DNase-resistant Z conformation or becomes protected by interactions with other matrix components, such as curli.

Next, we used a previously established chemical approach to shift eDNA toward specific conformations, applying chloroquine to favor the B-DNA conformation and Cerium (III) Chloride (CeCl₃) to promote the Z-DNA conformation [33,34] (Fig 2D-2F). We initiated biofilm formation and added chloroquine or CeCl₃ 24 hours after biofilm establishment, then examined biofilm architecture at 96 hours using confocal microscopy and Comstat quantitation. Both treatments showed visual changes in the biofilm architecture. Untreated biofilms were visually homogenous in height with variable cellular packing (density). Chloroquine-treated (B-DNA-enriched) biofilms were visually more variable in height, and exhibited a modest reduction biomass and thickness compared to untreated controls, which did not reach statistical significance (Fig 2F). In contrast, CeCl₃-treated biofilms (Z-DNA-enriched) exhibited a more heterogeneous appearance, with denser bases and structures protruding above the base, however, again, analysis of the images by Comstat quantitation did not reveal statistically significant differences (Fig 2F). Comstat-quantified biomass reflects the thickness and surface area occupied by cells, but does not directly measure cellular density. In contrast, crystal violet-quantified biomass captures both cellular density and ECM density. Therefore, we also used the crystal violet assay, which quantifies the amount of stain absorbed to measure cellular density (Fig 2G-2H), extracellular components, and charged surfaces, to assess how chloroquine and CeCl₃ influence biofilm biomass when added at different stages of biofilm development. Chloroquine (B-DNA enrichment) was added at 0 h (inoculation) or at 24, 48, or 72 h after biofilm initiation and was maintained in the culture thereafter (Fig 2H). In all cases, chloroquine treatment significantly inhibited biofilm formation compared to untreated controls (Fig 2G). In contrast, continuous exposure to CeCl₃ (Z-DNA enrichment) resulted in increased biofilm biomass compared with control conditions (Fig 2G). Notably, even CeCl₃ treatment initiated at 48 or 72 h was sufficient to increase biofilm biomass relative to controls (Fig 2G). Together, these methods suggest that B-DNA enrichment by chloroquine reduces biofilm density, whereas Z-DNA enrichment by CeCl₃ increases it (visually and suggested by crystal violet biomass). It is important to note that CeCl₃ added at time 0, prior to the initiation of biofilm assembly, resulted in an increase in biofilm mass.

**Fig 3:**
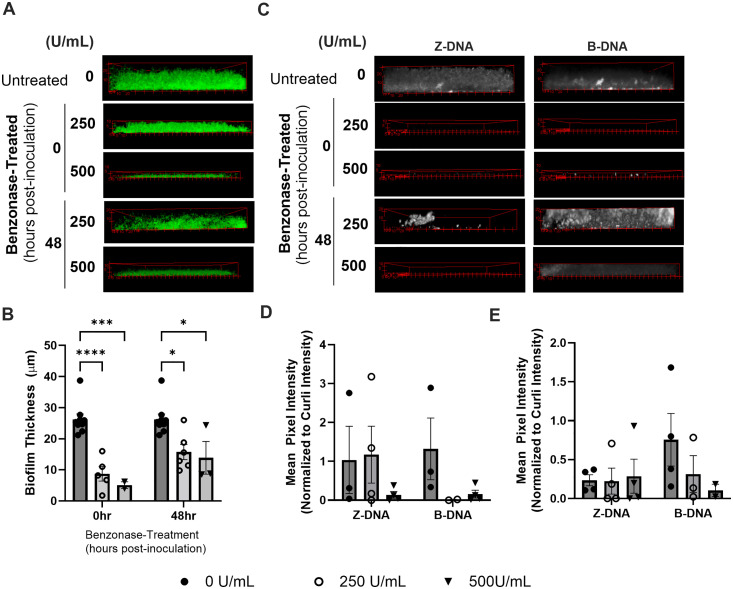
Degradation of Z-DNA and B-DNA reduces biofilm thickness. Biofilms were treated at setup (0 hours) or 48 hours post-inoculation with the indicated doses of benzonase. **(A)** Biofilms were stained with Syto9, imaged via confocal microscopy at 63X, and (C) biofilm thickness was measured. Biofilms treated with benzonase either (D) at setup (0 hours), or (E) 48 hours post-inoculation were stained for Z-DNA, B-DNA **(B)**, and curli, and MFI of Z-DNA and B-DNA relative to curli MFI was measured in ImageJ. The experiment was repeated at least three times. Representative images are shown. Each dot represents the average of three visual fields per one biofilm **(C-E)**.

We next examined how the degradation of extracellular nucleic acids affects STm biofilm structure using benzonase, a broad-spectrum endonuclease from *Serratia marcescens* that cleaves both DNA forms regardless of sequence or conformation [35,36]. Benzonase was applied to developing biofilms at increasing concentrations either at the onset of biofilm formation (0 hours) or after mature biofilms had formed (48 hours post-inoculation). When benzonase was added at the beginning of biofilm development, we observed a stronger reduction in biofilm formation (Fig 3A–3B), indicating that eDNA remained available for enzymatic degradation during the initial stages. In contrast, when benzonase is added at 48 hours, the effect is markedly reduced, suggesting that a fraction of the eDNA becomes protected from nuclease digestion, potentially through interactions with other ECM components.

To further examine the presence of Z-DNA and B-DNA, we used immunofluorescence to quantify both conformations present in the biofilm matrix following increasing doses (0 U/mL, 250 U/mL, and 500U/mL) of benzonase, at the onset of biofilm growth (0 hours) and after the biofilm was established (48 hours post-inoculation). In both early and established biofilms, benzonase reduced the abundance of extracellular Z-DNA and B-DNA (Fig 3C-3E), indicating that both canonical and non-canonical DNA structures are susceptible to enzymatic degradation within the matrix.

Taken together, these findings demonstrate that disruption of biofilm eDNA, including Z-DNA and B-DNA, compromises the structural integrity of STm biofilms. These results further support a model in which Z-DNA contributes to ECM stability, potentially through interactions with other ECM molecules, such as curli, and plays a previously unrecognized role in the maturation and resilience of *Salmonella* biofilms.

### Curli complexes with Z-DNA and induces anti-Z-DNA antibodies

**Fig 4:**
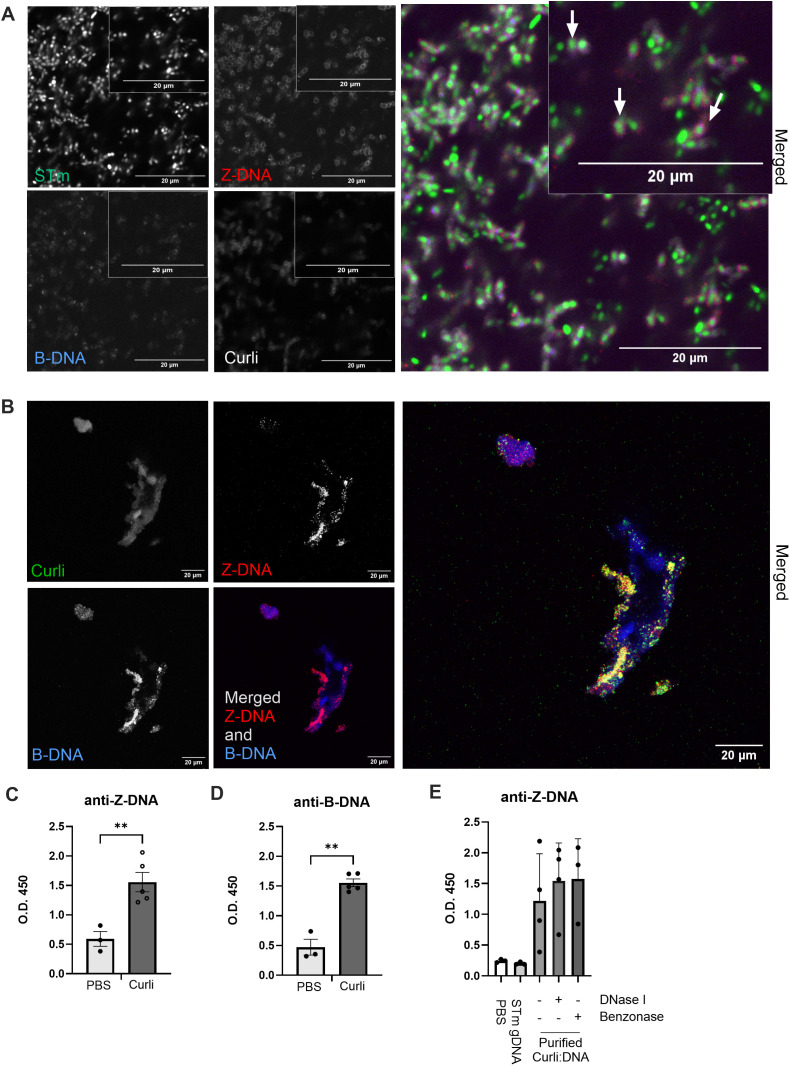
Curli complexes with Z-DNA and elicit anti-Z-DNA antibodies. **(A)** Biofilms were grown on glass cover slips and stained with Syto9 (green), anti-Z-DNA antibody (red), anti-B-DNA antibody (blue), and FSB amyloid stain for curli (gray). Images were acquired at 100X using confocal microscopy and merged in ImageJ. White arrows indicate examples of colocalization. **(B)** Purified curli (green) isolated from *in vitro* 72-hour STm biofilms was stained for both Z-DNA (red) and B-DNA (blue). **(C-D)** C57BL/6 mice were i.p. injected with purified curli:DNA (100 µg), or sterile PBS for controls, weekly for 13 weeks and serum diluted 1:400 was analyzed by ELISA for (C) anti-Z-DNA [brominated poly(dGdC)] and (D) anti-B-DNA (calf thymus DNA) antibodies. **(E)** C57BL/6 mice were i.p. injected with genomic DNA extracted from STm; purified curli:DNA preparations containing an estimated 50μg curli and 45ng DNA treated with DNAse I, Benzonase, or untreated with nuclease post-purification; or PBS for controls, twice per week for 5 weeks. Each dot represents one mouse **(C-E)**. Serum was diluted 1:400 and analyzed by ELISA for anti-Z-DNA antibodies.

Curli forms amyloid-like fibrils that bind to extracellular bacterial DNA within the ECM of STm biofilms, and these curli:DNA complexes are substantially more immunogenic than either curli or DNA alone. To further define the nature of these complexes, we sought to determine whether curli fibrils preferentially associate with B-DNA, Z-DNA, or both within intact biofilms. To this end, we stained 3-day STm biofilms for B-DNA, Z-DNA, and curli. Consistent with prior observations, curli assembles into basket-like structures that encase individual bacterial cells, appearing as ring-like formations by microscopy [37]. As previously reported, the DNA signals were concentrated within these curli rings (Fig 4A). We detected a robust Z-DNA signal throughout STm biofilms, and curli co-localized with both B-DNA and Z-DNA in close proximity to bacterial cells. Notably, Z-DNA appeared tightly associated with curli fibrils within these basket-like structures, appearing as rings around the cells visible in the Z-DNA, whereas B-DNA was more frequently detected at the periphery of curli assemblies, occupying regions that were more exposed to the extracellular environment (Fig 4A). These spatial differences suggest that distinct DNA conformations may differentially interact with curli and contribute uniquely to biofilm architecture and stability.

When we purified curli from *in vitro* STm biofilms and used immunofluorescence to visualize DNA, we observed that curli purified from biofilms contains both Z-DNA and B-DNA (Fig 4B). Importantly, the curli purification process includes multiple rounds of DNase I and RNase treatment, boiling in sodium dodecyl sulfate (SDS), and overnight electrophoresis through a preparative SDS-polyacrylamide gel to remove contaminating nucleic acids. The persistence of DNA following these stringent conditions suggests that DNA is tightly bound to and protected by curli.

We have previously shown that intraperitoneal injection of purified curli containing DNA induces the production of antibodies against dsDNA and chromatin within two weeks. Here, we sought to determine whether administration of DNA-containing curli also promotes the generation of antibodies specific to distinct DNA conformations, namely B-DNA and Z-DNA. To address this question, mice were intraperitoneally injected with 100 µg of purified curli weekly for 13 weeks. To distinguish Z-DNA-specific antibodies, we employed the same experimental approach used in prior studies [38,39] to define anti-DNA antibody specificities in mouse sera. Serum antibody responses were assessed by ELISA by coating plates with calf thymus B-DNA and Z-DNA [brominated poly(dGdC)]. Injection of purified curli in mice induced robust antibody responses against both Z-DNA and B-DNA (Fig 4C and 4D).

Notably, in a separate experiment, intraperitoneal injection of STm genomic DNA alone did not result in the generation of anti-Z-DNA autoantibodies, whereas injection of purified curli without any adjuvant, which contains an apparent mixture of Z- and B-DNA, did, indicating that the curli scaffold is required to confer Z-DNA immunogenicity. Furthermore, pretreatment of purified curli with DNase I or benzonase did not reduce the magnitude of the anti-B-DNA or anti-Z-DNA antibody responses (Fig 4E). While DNase I has limited activity against Z-DNA, benzonase degrades DNA regardless of conformation. The lack of any reduction in anti-Z-DNA antibody responses following treatment with either nuclease suggests that DNA associated with curli complexes is physically protected from enzymatic access, limiting effective degradation. Consistent with this observation, DNA extracted from nuclease-treated curli preparations did not show a reduction in DNA concentration compared to untreated samples, indicating that these nucleases were ineffective at accessing the DNA within the curli complexes.

### STm genome exhibits a high propensity for Z-DNA formation

**Fig 5:**
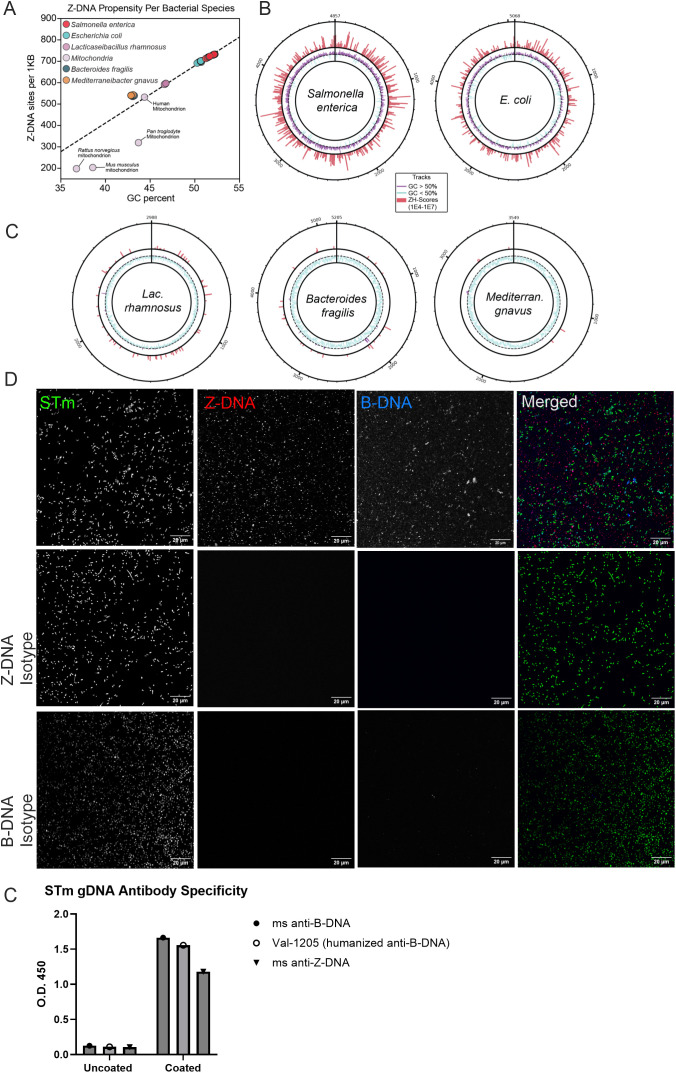
The STm genome contains regions in Z-configuration. **(A)** Five bacterial genomes and 4 mitochondrial genomes were analyzed using the ZHUNT algorithm, and plotted based on Z-DNA sites per 1KB (Y-axis) and GC% (X-axis). Genome-wide Z-DNA–forming potential visualized using ZDNABERT-filtered ZHUNT scores. Circos plots show **(B)** *Salmonella enterica* serovar Typhimurium str. LT2 and *Escherichia coli* str. K-12 substr. MG1655; and **(C)** *Bacteroides fragilis* NCTC 9343*, Lacticaseibacillus rhamnosus* NCTC 13764*,* and *Mediterraneibacter gnavus* ATCC 29149*.* The outer red track indicates ZH-scores ranging from 1 × 10⁴ to 1 × 10⁷, representing increasing Z-DNA-forming propensity. The inner track shows GC content, with regions >50% GC shown in purple and <50% GC shown in blue. **(D)** STm (green) was lysed during log phase growth, fixed, and stained for STm-exposed nucleic acids, including Z-DNA (red) and B-DNA (blue). **(D)** Plates were coated with STm gDNA, and binding of mouse (ms) anti-Z-DNA, mouse (ms) anti-B-DNA, and humanized anti-B-DNA (Val-1205) antibodies was analyzed by ELISA.

While our data demonstrate that Z-DNA is present in STm biofilms and accessible to immune recognition, its origin remains unclear. To address this question, we analyzed the STm genome using the previously established Z-HUNT and Z-DNABERT algorithms, which predict the energetic favorability of DNA sequences to adopt the left-handed Z-DNA conformation [40]. This analysis revealed that the STm genome exhibits a high number of ZH-sites per 1KB (~733–713), indicating a strong intrinsic tendency to form Z-DNA structures (Fig 5A, 5B). These results are consistent with the GC-rich nature of the *Salmonella* genome (~52.3% GC content [41,42] 44.3%-36.7% for human, mouse, rat, or ape mitochondrial genomes [43]). Notably, the genome of *E. coli,* in the same Enterobacteriaceae family as *Salmonella*, has a similarly high GC content (~50.6%), and also showed a high Z-DNA-forming potential (Fig 5A,5B). In contrast, other common gut commensals, including *Bacteroides fragilis* and *Lactobacillus rhamnosus*, showed substantially lower Z-DNA-forming potential (Fig 5A, 5B), with 43.1% and 46.6% GC content, respectively. These results together show a strong correlation between GC-rich genomes and the propensity to form Z-DNA (Spearman’s ρ = 0.98), a trend well established [44,45]. Interestingly, *Mediterraneibacter gnavus* (formerly *Ruminococcus gnavus*), a bacterium whose expansion in the gut of SLE patients also positively correlates with disease severity [44] did not exhibit highly Z-prone genome or contain a GC-rich genome (42.8%), suggesting that Z-DNA propensity is not a universal feature of inflammation-associated microbes.

To experimentally validate whether STm genomic DNA (gDNA) contains regions capable of adopting the Z conformation, we lysed STm cells during logarithmic growth, fixed the lysates to maintain supercoiling, and stained the exposed genomic DNA with Syto9 or antibodies specific for Z-DNA and B-DNA. As these experiments indicated, both Z- and B-DNA-specific antibodies robustly bound to the lysed STm genome, indicating the presence of both DNA conformations within bacterial chromosomal DNA (Fig 5D). Consistent with these findings, both anti-Z-DNA and anti-B-DNA antibodies bound efficiently to purified STm gDNA, as tested by coating plates with STm gDNA and analyzing antibody binding via specific ELISA assays (Fig 5E). Together, these findings demonstrate that the STm genome contains intrinsic sequence features that favor Z-DNA formation, supporting the conclusion that bacterial genomic DNA is a plausible source of the Z-DNA detected within curli-associated biofilm complexes.

### *In vivo* STm infection induces the generation of anti-Z-DNA antibodies

**Fig 6:**
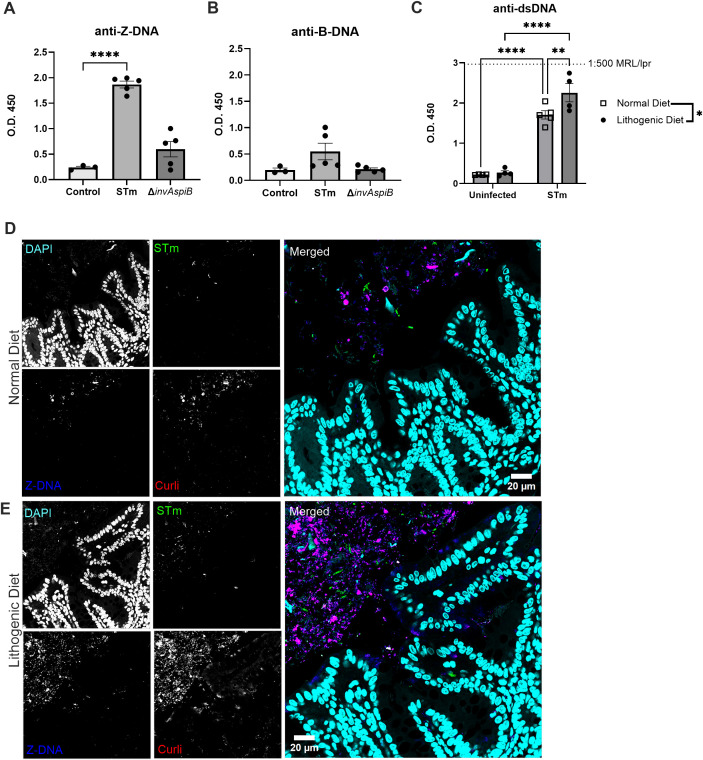
A lithogenic diet induces Z-DNA-containing STm biofilms and higher anti-DNA antibodies. **(A-B)** 129X1/SvJ mice were intragastrically infected with STm IR715 or an isogenic non-invasive mutant STm (*invAspiB*). The serum was collected 13 weeks post-infection, diluted 1:600, and assayed by ELISA for (A) anti-Z-DNA and (B) anti-B-DNA antibodies. **(C-E)** 129X1/SvJ mice were fed a lithogenic (high-cholesterol) diet for 8 weeks, then switched to a normal diet for 2 weeks before i.p. infection with STm. **(C)** Serum from 3 weeks post-infection was diluted 1:250 and analyzed by ELISA for anti-dsDNA autoantibodies. Each dot represents one mouse. **(D-E)** Cecal tissue was stained with antibodies against *Salmonella* (green) and Z-DNA (blue), EBBA Biolight 680 bacterial amyloid dye for curli (red), and DAPI for host nuclei (cyan). Representative images were taken at 63X magnification on the confocal microscope and processed via ImageJ.

To investigate how a STm infection affects the generation of Z-DNA antibodies, we orally infected 129X1/SvJ mice with STm and followed the mice for 13 weeks. 129X1/Sv mice are commonly used in *Salmonella* infection studies because they carry a functional SLC11A1, which allows control of bacterial growth while permitting long-term persistence. As a result, they develop a sustained, systemic infection that serves as a well-established model of persistent Salmonellosis [46]. Mice infected with wild-type STm developed robust anti-Z-DNA antibody responses. In contrast, infection with a non-invasive *invAspiB* mutant failed to induce detectable anti-Z-DNA antibodies (Fig 6A). Notably, wild-type STm infection also appeared to elicit anti-B-DNA antibodies (Fig 6B). Together, these findings demonstrate that, once bacteria breach the intestinal epithelial barrier, invasive STm infection may drive a strong and preferential anti-Z-DNA antibody response.

Recent work has shown that a high-cholesterol lithogenic diet (LD; 1% w/w cholesterol, 0.5% cholic acid), commonly used to induce hypercholesterolemia in mice, enhances STm biofilm formation in the gut, which correlates with elevated cecal cholesterol level [20]. To determine whether increased biofilm formation influences autoantibody responses, 129X1/SvJ mice were fed LD for 6 weeks, then switched to normal chow (normal diet, ND) for 2 weeks prior to i.p. infection with wild-type STm. LD-fed mice developed significantly stronger anti-dsDNA autoantibody responses than mice fed ND (Fig 6C). Consistent with these serologic findings, histological analysis of intestinal sections from LD-fed mice revealed that STm biofilms exhibited an abundance of Z-DNA in LD-fed mice compared to ND-fed mice (Fig 6D-6E). These data support the idea that diet-induced changes in the abundance of biofilm matrix molecules and exposure to immunostimulatory Z-DNA and promote autoantibody production

## Discussion

Infection has long been suspected to contribute to the induction of anti-DNA antibodies, yet the underlying mechanisms remain incompletely defined. The findings presented here demonstrate that bacterial biofilms, specifically biofilm-associated DNA complexed with curli, constitute a potent, structurally distinct source of immunogenic DNA that drives anti-DNA antibody production. These data indicate that both bacterial DNA and bacterial products such as curli can promote anti-DNA responses in normal immunity and may contribute to the pathogenesis of SLE, the human disease most strongly associated with aberrant immune responses to DNA [3,38,39,45]. Anti-DNA antibodies are observed in multiple autoimmune diseases, including SLE, rheumatoid arthritis, Sjögren’s syndrome, and autoimmune hepatitis [47]; however, in SLE they correlate particularly well with disease activity and clinical flares. Indeed, SLE is considered the prototypic autoimmune disease in which anti-DNA antibodies serve as a defining serologic hallmark. These antibodies display features of antigen-driven selection, including somatic hypermutation and affinity maturation, consistent with a sustained and specific immune response to DNA-containing antigens [45,48]. Although host-derived DNA released during apoptosis and necrosis has long been considered a major source of immunostimulatory nucleic acids in SLE [49,50], our findings identify bacterial biofilms as an additional, structurally distinct reservoir of immunologically active DNA.

Our findings provide a useful perspective on the roles of both infection and the microbiome in lupus pathogenesis. Several studies have now reported that Pseudomonadota (formerly Proteobacteria), the dominant phylum of Gram-negative bacteria, which includes the *Enterobacteriaceae* family containing *Salmonella* and *E. coli*, as well as the Gram-positive *Mediterraneibacter* (formerly *Ruminococcus*) are overrepresented in the intestinal microbiota of patients with active SLE [51–53]. However, the mechanisms by which these bacteria contribute to pathogenesis remain largely unknown. Using STm as a model organism, we provide evidence for a unique mechanism by which Proteobacteria-derived biofilms may promote SLE-relevant immune responses to DNA, supporting the role of both foreign and self DNA as triggers for autoantibody production.

Using *in vitro* model systems, our studies demonstrate that STm biofilms contain abundant eDNA in both the canonical B-DNA and non-canonical Z-DNA conformations, with levels increasing as the biofilm matures (Fig 1). Under optimal biofilm-forming conditions, curli expression is upregulated over time and reaches maximal levels at approximately 48 hours, coinciding with advanced matrix development. eDNA is a well-established structural component of the biofilm ECM, contributing to biofilm integrity and stability. Importantly, our functional analyses reveal that both B-DNA and Z-DNA are required to establish biofilm architecture (Figs 2 and 3), indicating that the transition of DNA into the Z-conformation actively contributes to biofilm structure rather than representing a passive byproduct of biofilm maturation. Within the ECM, DNA is highly organized and complexed with matrix-associated proteins. Previous work has shown that DNABII family proteins play a critical role in the initial stabilization and organization of eDNA in biofilms; however, DNABII-associated DNA remains sensitive to nuclease degradation [31,54–57]. Although the precise role of curli in relation to biofilm DNA has not been fully defined, our data support a model in which curli interacts with DNA following its initial organization by DNABII proteins, thereby further stabilizing the biofilm matrix. Notably, the β-sheet motif of curli monomers organizes DNA into a spatially periodic lattice that amplifies immune activation [26]. In addition, DNA enhances curli polymerization, indicating a reciprocal interaction that promotes amyloid scaffold assembly within the ECM. Once curli is polymerized and incorporated into the matrix, DNA associated with curli becomes markedly more resistant to nuclease degradation (Figs 3B–3D, 4E), demonstrating that the amyloid scaffold both reinforces and protects the ECM.

Consistent with this model, curli-associated DNA is displayed to the immune system in a highly organized manner, forming an ordered and multivalent array of parallel dsDNA. This structured presentation enhances engagement with innate immune receptors, including TLR9, thereby promoting immune activation [26]. Curli:DNA complexes, but not purified genomic STm DNA alone, induced antibody responses *in vivo* (Fig 4C-4D). Although these complexes contain both B-DNA and Z-DNA (Fig 4B), i.p. injection elicited both anti-B-DNA and anti-Z-DNA autoantibodies, demonstrating that curli association is essential for DNA immunogenicity. Notably, although *in silico* analysis using the Z-Hunt algorithm predicts that the GC-rich STm genome is highly prone to adopting the Z-DNA conformation, genomic DNA alone failed to induce anti-Z-DNA antibodies (Figs 4E, 5A). These findings underscore the importance of curli-mediated stabilization and presentation of Z-DNA for elicitation of an effective anti-Z-DNA antibody response. In this context, curli likely functions as both a carrier and a structural scaffold, converting DNA from a normally inert molecule into a potent danger signal. We have previously shown that curli:DNA complexes engage both TLR2/TLR1 and TLR9 signaling pathways [23,58–61], further supporting a role for curli as a delivery system that promotes innate immune sensing and downstream autoimmune responses.

Importantly, differences in GC composition across bacterial genera influence TLR9 activation, as this receptor specifically recognizes unmethylated CpG-containing DNA motifs. Indeed, prior analyses of DNA from multiple bacterial species demonstrated variable TLR9-activating capacity, with *E. coli* exhibiting both the highest CpG content and the strongest activation [62]. Beyond CpG frequency alone, genomic propensity to adopt alternative DNA conformations may further modulate immunogenicity. Comparative Z-Hunt analyses revealed that STm and closely related *E. coli* genomes are highly enriched in regions with a propensity to adopt the Z-DNA conformation, whereas *R. gnavus*, a gut commensal associated with SLE, exhibits a lower Z-DNA–forming potential (Fig 5A). This analysis suggests that members of the phylum Proteobacteria may be inherently better able to generate Z-DNA–rich extracellular matrices compared to other commensals and *R. gnavus*, particularly when DNA is present in curli-containing biofilms. As a result, Proteobacteria-derived biofilms may represent a more potent source of immunogenic Z-DNA than those of *R. gnavus*, thereby driving more effective anti-Z-DNA antibody responses in SLE patients. Together, these findings support a model in which the taxonomic composition of the gut microbiota, including the enrichment of Z-DNA–prone Proteobacteria, influences an array of microbial signals that act on the immune system and drive anti-DNA production. Extending these observations *in vivo*, we find that invasive STm infection induces anti–B-DNA antibodies; however, these responses are modest and significantly lower than those directed against Z-DNA. In contrast, non-invasive STm infection confined to the intestinal lumen fails to elicit anti–DNA antibody responses (Fig 6A), indicating that luminal exposure alone is insufficient to break immune tolerance. These data demonstrate that invasive STm infection, when bacteria breach the intestinal epithelial barrier and gain access to host immune cells, drives a robust and preferential anti–Z-DNA response [63,64].

To further elucidate the amplification of these responses by host factors (e.g., diet), we employed a lithogenic (high-cholesterol) diet that promotes bile acid–dependent biofilm formation in the cecum [20]. Use of this diet resulted in a marked increase in anti-DNA antibody production during STm infection (Fig 6C), indicating that diet-enhanced biofilm formation can amplify systemic anti-DNA responses. In LD-fed mice, STm biofilms extended closer to the epithelium and exhibited significantly greater Z-DNA abundance compared to mice fed a standard diet (Fig 6D-6E). These findings suggest that dietary modulation of the gut environment can promote biofilm formation and boost the immune responses to biofilm-associated molecular patterns.

Beyond SLE, invasive enteric infections, including STm, are clinically associated with reactive arthritis (ReA) [65–71]. While most individuals experience self-limiting gastroenteritis, a subset, particularly those carrying the HLA-B27 genotype, develop chronic inflammatory arthritis weeks after infection [72,73,74]. In mouse models, STm biofilms and curli produced in the intestine play critical roles in joint inflammation and synoviocyte hyperplasia [18], further supporting the idea that immune signaling by biofilm molecules can drive chronic inflammation. A relevant question is whether antibody responses to DNA are similarly observed in patients with ReA and whether these antibodies contribute to the disease.

In addition to demonstrating the influence of infection on anti-DNA production, this study shows that dietary changes can alter the organization of the gut microbiota and its biofilm-forming capacity, thereby affecting the quality and quantity of microbial signals that influence the immune system. By promoting Z-DNA-rich biofilms, dietary factors, such as a lithogenic diet, may amplify anti-DNA antibody responses. In the future, it will be critical to determine whether bacterial biofilms and biofilm-derived nucleic acid/protein complexes play a broader role in promoting inflammation in SLE and other conditions, potentially linking intestinal dysbiosis, autoantibody production, and systemic inflammation.

## Materials and methods

### Ethics statement

Experiments were performed under protocols approved by Association for Assessment and Accreditation of Laboratory Animal Care (AAALAC)-accredited Temple University Lewis Katz School of Medicine and Nationwide Children’s Hospital, Institutional Animal Care and Use Committee (IACUC), on file with the National Institutes of Health (NIH) Office for the Protection of Research Risks, in accordance with United States Department of Agriculture (USDA) and Public Health Service (PHS) Policy on Humane Care and Use of Laboratory Animals.

### Bacterial growth

*S.* Typhimurium strain 14028 and *S.* Typhimurium strain IR715 were used in this study. *S.* Typhimurium strain IR715 is a fully virulent, nalidixic acid-resistant strain derived from the ATCC strain 14028 [75]. The SPI-1/SPI-2 type three secretion-negative IR715 Δ*invAspiB* mutant was described previously [76,77]. Single colonies of STm were inoculated into 5 mL Luria Broth (LB) supplemented with 50 μg/mL nalidixic acid or Tryptic Soy Broth (TSB) and grown overnight at 37°C with shaking.

### *In vitro* biofilms

Sterile glass coverslips were placed into a 24-well Fisherbrand Tissue Culture Plate (FB012929), and the surrounding wells were filled with sterile water to prevent drying. STm cultures grown overnight were diluted 1:100 in LB without salt (LBNS) to 300 μL per well. The plate was covered and incubated statically at a 45° angle in a 28°C incubator for each time point (72 hours or 7 days) without media changes. The supernatant was discarded, and each well was gently washed three times with sterile PBS (sPBS). Biofilms were blocked for 1 hour with a 1:200 dilution of Anti-CD16/CD32 FC Shield Antibody and then washed with sPBS. Biofilms were incubated at room temperature for 1 hour with primary antibodies (5μg/mL), washed, then incubated for 1 hour with 1:200 dilution of secondary antibodies. Slides were incubated at room temperature for 15 minutes with Syto9 Green Fluorescent Nucleic Acid Stain (0.3%), then washed with sPBS. Glass coverslips were carefully removed from wells and placed top-down on an 8-well microscope slide containing a drop of Vectashield. Coverslips were sealed with clear nail polish and imaged at 100X using a Leica SP5 confocal microscope. The following commercial antibodies were used for immunofluorescence: 5μg/mL rabbit IgG isotype control (AC042), 5μg/mL mouse IgG2b isotype control (02–6300), 5μg/mL mouse anti-Z-DNA/anti-Z-RNA antibody [Z22] (Absolute Antibodies Ab00783-3.0) (Z-DNA), 5μg/mL rabbit anti-dsDNA antibody [DSD/4054R] (Novus Biologicals NBP3-07302-100ug) (B-DNA). The following secondary antibodies were used in a 1:200 dilution: AlexaFluor555-conjugated AffiniPure donkey anti-mouse IgG (H + L) (0.50mg) (Jackson Immuno Research 715-565-150) and goat anti-rabbit IgG H&L (Alexa Fluor 647) (Abcam ab150079).

STm biofilms were grown in LBNS and treated with varying concentrations of benzonase (0U/mL, 250U/mL, 500U/mL) at the beginning of biofilm growth (0 hours) or after maturation (48 hours post-inoculation). Biofilms were stained at room temperature for 15 minutes with Syto9 Green Fluorescent Nucleic Acid Stain and mounted and imaged as described above. Mean pixel intensity was calculated by first generating an average-intensity projection of each channel’s Z-stack in ImageJ, then measuring the mean pixel intensity of the resulting image. These values were normalized by dividing by the mean pixel intensity of the curli channel.

STm biofilms grown in 1:20 TSB media were treated with DNase (10U/mL, ThermoFisher), chloroquine (0.5 mM, Sigma Aldrich), or CeCl_3_ (0.5 mM; gift from the Goodman Lab at Nationwide Children’s Hospital). DNAse, chloroquine, or CeCl_3_ were added with the media and the inoculum (0 hours), or at 24 hours, 48 hours, or 72 hours post-inoculation, before analysis at 96 hours. Media containing the appropriate treatment was replaced daily. After 96 hours, the biofilms were washed once with PBS. Cells were labeled with Syto60 (5 mM; Molecular Probes) in 5% bovine serum albumin (BSA) blocking buffer at room temperature for 30 minutes, after which time the stain was carefully removed and discarded. The wells were washed three times with PBS before the addition of 200 µL 4% paraformaldehyde (PFA; Affymetrix) at room temperature for 20 minutes. Stained biofilms were visualized, and three-dimensional biofilm images were acquired by capturing 2 random Z-stacks per well, 3 wells per treatment, using a Zeiss LSM 800 confocal laser scanning microscope at 63X magnification. The Z-stacks were then analyzed using the software package Comstat2 to calculate biomass as 3D volume (μm^3^) over area (μm^2^) and average thickness (μm) for the entire area.

### *In vitro* biofilms for crystal violet assay

A single colony *S.* Typhimurium 14028 was grown in 5 mL TSB at 37°C overnight on a roller drum. The overnight culture was normalized to OD_600_ = 0.47 (optical density at 600 nm) and diluted 1:2500 in 1:20 TSB. After diluting, 200 µL of the diluted culture was added to a non-treated polystyrene 96-well plate in quadruplicate. The plate was incubated for 96 hours at 25°C statically, with 1:20TSB media containing the appropriate treatments (untreated, 0.5mM chloroquine, 0.5mM CeCl_3_) replaced every 24 hours. At 96 hours, the plate was washed twice with double-distilled water (ddH_2_O) and heat-fixed for 1 hour at 60°C. The plate was stained with 0.33% crystal violet solution for 5 minutes at room temperature. After two washes with ddH_2_O, 100 µL of 33% acetic acid was added. The OD_570_ of was measured in a SpectraMax spectrophotometer with SoftMax Pro software (Molecular Devices) to determine the remaining amount of crystal violet stain. This was performed in triplicate.

### Curli purification

Curli aggregates were purified from the *S.* Typhimurium IR715 *msbB* mutant as previously described [78]. After the purification steps, curli preparations were then resuspended in sterile water. Concentrations of curli aggregates were determined using the bicinchoninic acid (BCA) assay according to the manufacturer’s instructions (Novagen, 71285–3). Curli protein preparations were adjusted to be 1 mg/mL, aliquoted and stored frozen at -20°C.

### Immunofluorescent imaging of purified curli

8-well microscope slides were coated for 1 hour with 50ul poly-L-lysine. The slides were washed with sPBS, and 50 μL of 1 mg/mL curli protein preparation was added to each well, and incubated overnight at 4°C. Wells were washed with sPBS and incubated with the antibodies named below. Glass coverslips were placed on top of each well and were sealed with clear nail polish and imaged using a Leica SP5 confocal microscope.

The following commercial antibodies were used: 5μg/mL rabbit IgG isotype control (AC042), 5μg/mL mouse IgG2b isotype control (02–6300), 5μg/mL mouse anti-Z-DNA/anti-Z-RNA antibody [Z22] (Absolute Antibodies Ab00783-3.0) (Z-DNA), 5μg/mL rabbit anti-dsDNA antibody [DSD/4054R] (Novus Biologicals NBP3-07302-100ug) (B-DNA). The following secondary antibodies were used in a 1:200 dilution: AlexaFluor555-conjugated AffiniPure donkey anti-mouse IgG (H + L) (0.50mg) (Jackson Immuno Research 715-565-150) and goat anti-rabbit IgG H&L (Alexa Fluor 647) (Abcam ab150079).

### Treatment of mice with purified curli

Male and female C57BL/6 (wild type) mice were purchased from Jackson Labs at 4–6 weeks old. At 6–8 weeks of age, mice were injected intraperitoneally (i.p.) with 100 µg of curli:DNA complex or sterile PBS (control) once per week, alternating sides for 13 weeks. After euthanasia with CO_2_, mice were exsanguinated and the blood was collected for analysis.

For the experiments using nuclease-treated purified curli, aliquots of 50 µg curli:DNA complexes were either untreated (control) or incubated at 37°C for 30 minutes with 500 U/mL DNase I or 500 U/mL Benzonase (Sigma-Aldrich, 9025-65-4). Treatments were then incubated at 80°C for 30 minutes to denature the nucleases. Mice were i.p. injected with 100 µL PBS, 45 ng STm genomic DNA as controls, or 50 µg of curli:DNA preparations twice per week for 5 weeks [26].

### Anti-DNA ELISA

The ELISA to quantify anti-dsDNA antibodies was performed as previously described [38,39]. Z-DNA and B-DNA antigens were obtained and prepared as previously described [38,79]. Briefly, poly(dGdC) was brominated (Br-poly(dGdC)) to serve as the Z-DNA antigen according to the following protocol: poly(dGdC) was reconstituted with TE buffer, the sodium chloride content of an aliquot of this stock was adjusted to 150 mM, and the stock was then diluted to 200 μg/mL with citrate/EDTA/NaCl buffer. Bromine water, diluted 1:25 with UltraPure Distilled Water, and poly(dGdC) were mixed in a 1:3:1 ratio and incubated for 20 minutes in the dark at room temperature. Commercially available calf thymus DNA (Sigma-Aldrich) served as the B-DNA antigen.

ELISA assays were performed as described previously [38]. Briefly, plates were coated overnight with 100 μL/well of various DNA antigens (2 μg/mL) diluted in Saline-Sodium Citrate (SSC) Buffer. Control wells had SSC alone. Coated plates were incubated overnight at 4 °C. The next day, plates were washed with PBS, followed by blocking for 2 hours at room temperature with blocking buffer (2% bovine serum albumin (BSA), 0.05% Tween-20 in PBS). After blocking, the plates were washed with PBS and incubated for 1 hour at room temperature with horseradish peroxidase (HRP)-conjugated secondary reagent: anti-mouse IgG (γ chain specific [EMD Millipore]) at 1:1000; anti-sheep IgG (H + L chain specific [EMD Millipore]) at 1:1000; or anti-human IgG (γ chain specific [Sigma-Aldrich]) at 1:1500. The reaction proceeded for 1 hour at room temperature. The secondary antibodies were diluted with PBS ELISA dilution buffer (P-EDB; 0.1% BSA, 0.05% Tween 20 in PBS, pH 7.4). The plates were washed, then incubated with TMB substrate for 30 minutes at room temperature. Sulfuric acid was then added to terminate the color development. The absorbance was measured at 450 nm using a UVmax multi-plate spectrophotometer (Molecular Devices).

### Infection of mice

6-8 week-old 129X1/SvJ mice, including both males and females, were inoculated intragastrically with 20 mg of streptomycin (0.1ml of a 200 mg/mL solution in PBS) 24 hours before bacterial inoculation to induce gastrointestinal pathology [80]. Bacteria were grown shaking in LB broth at 37°C overnight. Mice were inoculated intragastrically with either 0.1 mL of sterile LB broth (mock infection) or 10^7^ − 10^8^ CFU *S.* Typhimurium IR715 or the *invAspiB* mutant.

For LD experiments, 6–8 week old male 129X1/SvJ mice were fed a high cholesterol (1% cholesterol, 0.5% cholic acid, Envigo, TD 140673; lithogenic) diet for 6 weeks. After this time, mice were taken off the high-cholesterol diet and put on a normal diet for 10 days. Mice were infected i.p. with 2 x 10^3^ STm strain 14028, without streptomycin pre-treatment. For the Z-DNA staining in cecal tissue, 6–8 weeks old male and female 129X1/SvJ mice were fed a lithogenic diet and then intraperitoneally infected with 1 × 10^^3^ CFU of STm 14028 and followed for 3 weeks. The 3-week time point was chosen because anti-DNA antibodies are generated within 2–3 weeks post-infection; the 13-week samples derive from an earlier experiment conducted prior to this understanding. Cecal tissue was collected, fixed in 10% formalin for 48 hours, then paraffin-embedded, and tissue sections were stained as previously described. Cecal tissue sections were stained with primary antibodies rabbit *Salmonella* O antiserum (1:500, Difco, 226591) and mouse anti-Z-DNA (1:200, Z22, Absolute Antibodies) or EBBA Biolight 680 bacterial amyloid dye (1:200), and secondary antibodies AlexaFluor488-conjugated goat anti-rabbit IgG (1:250, Life Technologies, A11008) and Rhodamine Red X-conjugated AffiniPure donkey anti-mouse IgG (H + L) (1:100, Jackson Immuno Research, 715-295-150). Sections were imaged at 63X magnification on the Leica SP5 confocal microscope and images were processed on ImageJ2.

### Z-HUNT algorithm

To evaluate the thermodynamic propensity of genomic regions to adopt the Z-conformation, we employed the Z-Hunt[rs] (ZH) program (v0.0.4, https://github.com/biomancy/zhuntrs), a Rust-based reimplementation of the original Z-Hunt algorithm [81] (v3), together with Z-DNABERT, a deep-learning model trained to identify Z-DNA-prone sequence contexts [82]. All available annotated type genomes for each species were obtained from NCBI GenBank, and “ZH-scores” were predicted across each chromosome using fixed-size windows (12–15 bp; corresponding to 6–7 dinucleotides). For the scatter plot visualization of selected bacterial and mitochondrial genomes, GC content was calculated as the percentage of guanine (G) and cytosine (C) bases in the total genome. Z-DNA site density was quantified as the number of Z-Hunt predicted sites per kilobase (sites/kb). Spearman rank correlation was calculated to assess the relationship between GC content and Z-DNA site density across all genome assemblies.

For Circos plot visualization of selected genomes, Z-DNABERT was run in conjunction with ZHUNT using the publicly available HG-Kouzine model to filter for highly Z-prone sequences. The genome was scanned in small overlapping 6-base segments, and the model’s predicted Z-DNA scores were compiled across the chromosome. Regions with consistently high scores (≥0.9) were marked and saved, and only stretches at least 12 bp long were retained as Z-prone DNA candidates.

### STm lysis and nucleic acid staining

A 5 mL overnight STm culture in LB supplemented with 50 μg/mL nalidixic acid was pelleted by centrifugation at 4000 x g for 5 minutes, resuspended in 1 mL 4% formaldehyde in PBS, and incubated at RT for 30 minutes. The pellet was then washed three times in PBS. After the final centrifugation, the supernatant was removed, and the pellet was resuspended in 70% ethanol and incubated for 1 hour at room temperature with shaking. To immobilize cells, an 8-well slide was coated with poly-L-lysine for 1 hour and washed three times with PBS prior to addition of cells. Cells were mixed with 1:1 volume water, centrifuged at 600 x g for 5 minutes, resuspended in PBS and mounted to pre-coated slide wells. Cells were incubated with 1 mg/mL lysozyme in Tris-EDTA-Glycine buffer for 30 minutes at room temperature. Staining and imaging of the nucleic acids were performed as described.

### STm genomic dna antibody specificity

STm genomic DNA was extracted following the instructions of the E.Z.N.A Bacterial DNA Extraction Kit (Thomas Scientific, C755C96). Antibody specificity was determined via ELISA, as described above.

### Statistical analysis

Data were analyzed using Prism software (GraphPad). Two-way ANOVA with post-hoc Tukey multiple comparisons tests or two-tailed Student’s t test were used as appropriate. The p-values <0.05 were considered significant. *p < 0.05, **p < 0.01, ***p < 0.001 were marked in the figures.

## Supporting information

S1 Data All raw data are available in the supporting information. (XLSX)
